# An Unusual Occurrence of Erythrocytosis in a Child with Nephrotic Syndrome and Advanced Chronic Kidney Disease

**DOI:** 10.3390/pediatric13030053

**Published:** 2021-08-04

**Authors:** Ratna Acharya, Kiran Upadhyay

**Affiliations:** 1 Division of General Pediatrics, Department of Pediatrics, University of Florida, Gainesville, FL 32610, USA; racharya@ufl.edu; 2 Division of Pediatric Nephrology, Department of Pediatrics, University of Florida, Gainesville, FL 32610, USA

**Keywords:** erythrocytosis, nephrotic syndrome, child, chronic kidney disease

## Abstract

**Background:** Anemia is common in patients with nephrotic syndrome (NS) for various reasons. Furthermore, anemia can occur in patients with chronic kidney disease (CKD) predominantly owing to inappropriately low erythropoietin (EPO) production relative to the degree of anemia. However, erythrocytosis is uncommon in patients with NS and advanced CKD who are not treated with exogenous erythropoietin stimulating agents, and when present, will necessitate exploration of the other etiologies. **Case summary:** Here, we describe an 8-year-old girl with erythrocytosis in association with NS and advanced CKD. The patient was found to have erythrocytosis during the evaluation for hypertensive urgency. She also had nephrotic range proteinuria without edema. Serum hemoglobin and hematocrit were 17 gm/dL and 51%, respectively, despite hydration. Renal function test showed an estimated glomerular filtration rate of 30 mL/min/1.73 m^2^. There was mild iron deficiency anemia with serum iron saturation of 18%. Serum EPO level was normal. Urine EPO was not measured. Renal biopsy showed evidence of focal segmental glomerulosclerosis. Genetic testing for NS showed mutations in podocyte genes: NUP93, INF2, KANK1, and ACTN4. Gene sequence analysis of genes associated with erythrocytosis showed no variants in any of these genes. She required chronic dialysis ten months later and, subsequently, a renal transplantation 14 months after the initial presentation. **Conclusion:** Since the serum EPO level was normal, an increased sensitivity to EPO is the most probable mechanism of erythrocytosis. The unusual association of erythrocytosis in patients with NS and advanced CKD needs to be studied further in larger studies.

## 1. Introduction

The hematocrit >51% in males and >48% in females is usually considered above normal and raises the possibility of erythrocytosis [1]. The raised red cell mass >25% above predicted value is also considered as erythrocytosis. Although anemia can occur in patients with nephrotic syndrome (NS) and chronic kidney disease (CKD), erythrocytosis is uncommon. Balal et al. described a 20-year-old female with minimal change disease who presented with erythrocytosis [2]. Nagaraju et al. described a 27-year-old male with minimal change disease associated with cerebral venous thrombosis [3]. Thrombotic episodes are common in nephrotic states and erythrocytosis can be a concurrent finding [4]. Erythrocytosis with elevated serum and urine erythropoietin (EPO) levels was described in a 23-year-old male with focal segmental glomerulosclerosis (FSGS) [5]. Similarly, Myers et al. described another case of true erythrocytosis associated with FSGS [6]. An increased serum EPO level has been described in patients with CKD and in hemodialysis [7,8]. Nistico et al. described an adult patient with obstructive sleep apnea and on hemodialysis who was found to have erythrocytosis and elevated serum EPO level [8].

Here, we report a child with NS secondary to familial FSGS with associated advanced CKD who presented with erythrocytosis. We report details of the clinical presentation and possible etiologies of the erythrocytosis.

## 2. Materials and Methods

This is a retrospective case study and the family of the patient provided consent for the study.

## 3. Case Presentation

An eight-year-old female of South Indian descent was presented to the emergency department for evaluation of headaches. She had headaches for several days, which were managed with acetaminophen at home. She denied history of blurry vision, dizziness, sleep apnea, syncope, and head trauma. There was no history of recent sore throat, skin infection, vomiting, and diarrhea. In the past, she had headaches of similar nature on an intermittent basis for about a year. There was no history of worsening of headaches with light or sound. Urine output was normal and there was no history of swelling of feet or abdomen or facial puffiness. There were no other known significant past medical problems including congenital heart disease. There was no history of administration of erythropoietin stimulating agents or recent travel to high altitude. She was born full term with no perinatal complications. Family history was significant for parental consanguinity, who were the first cousins. There was no family history of migraine headaches and erythrocytosis.

Upon examination, the vital signs showed an afebrile child with heart rate of 90 beats per minute, respiratory rate of 18 per minute, and manual blood pressure of 190/100 mm Hg, which remained persistently elevated upon repeat examinations. Her oxygen saturation was found to be low at 90–92% consistently, but did not require oxygen supplementation. The height was at the 75th centile and weight was at the 55th centile. Physical examination was remarkable only for strabismus. There was no periorbital puffiness, ascites, or pedal edema. She continued to have headaches. A non-contrast computed tomography scan of the brain showed no evidence of hemorrhage, infarction, or thrombosis. Hypertension was managed with intravenous hydralazine and labetalol. She was admitted for further evaluation of hypertension. Renal function test showed blood urea nitrogen of 14 mg/dL and serum creatinine of 1.6 mg/dL. Serum albumin was 3.1 gm/dL. The rest of the electrolytes were normal. Complete blood count showed hemoglobin 17 gm/dL, hematocrit 51%, white blood cell count of 7.2 × 10^9^/L, and platelet count of 247 × 10^9^/L (normal: 150–300 × 10^9^/L). Serum iron saturation was 18% (normal: 20–55%), iron was 45 µg/dL (35−150 µg/dL), transferrin was 176 mg/dL (200–360 mg/dL), total iron binding capacity (TIBC) was 246 µg/dL (225–430 µg/dL), and ferritin was 98 ng/mL. Serum vitamin B12 and folate levels were normal. Bone marrow biopsy was not obtained. Urinalysis showed 4 + proteinuria without microscopic hematuria. Random urine protein to creatinine ratio was 14.6. There was hypercholesterolemia. Serum complements were normal. Antinuclear, antineutrophil cytoplasmic, anti-glomerular basement membrane, and anti-double stranded DNA antibodies were negative. Hepatitis panel, human immunodeficiency virus, and tuberculin test were all negative. Thyroid function tests, serum catecholamines, plasma renin activity, and serum aldosterone were normal. Renal bladder sonogram showed bilateral echogenic kidneys with right kidney of 8.2 cm and left kidney of 8.7 cm in length with no hydronephrosis. Renal artery duplex study showed no evidence of renal artery stenosis. Chest X-ray showed no radiographic evidence of consolidation, pneumothorax, or effusion. Abdominal sonogram was normal. Nasopharyngeal swab for respiratory viruses were negative. Her oxygen saturation remained low for few days. Echocardiogram showed evidence of mild left ventricular hypertrophy, but no other abnormalities. Treatment consisted of intravenous hydration and initiation of antihypertensive agents, amlodipine, and labetalol. Blood pressures stabilized, but polycythemia was persistent (hemoglobin 15–16 gm/dL) (Table 1). Work-up for polycythemia included normal serum EPO (11 mU/mL; normal: 4–27 mU/mL) and normal gene sequence analysis of nine gene variants associated with erythrocytosis (genes tested: *BPGM, EGLN1, EPAS1, EPOR, HBA1, HBA2, HBB, JAK2,* and *VHL*).

**Figure 1:**
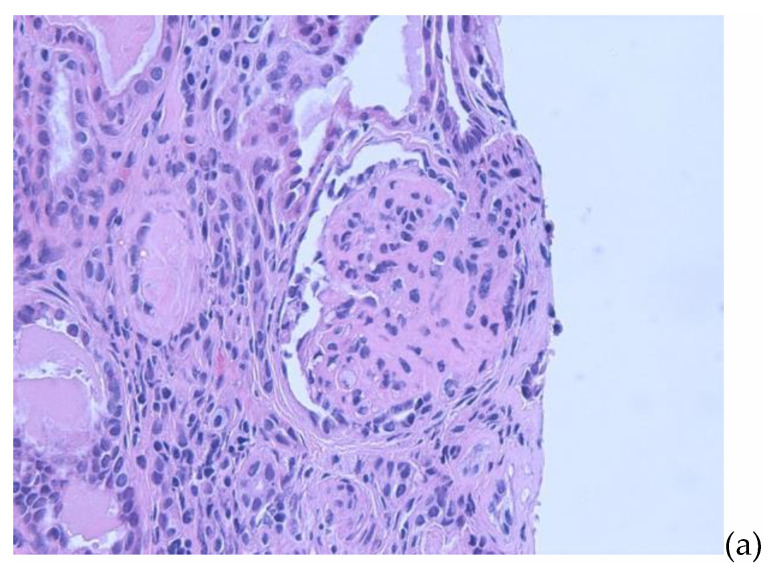

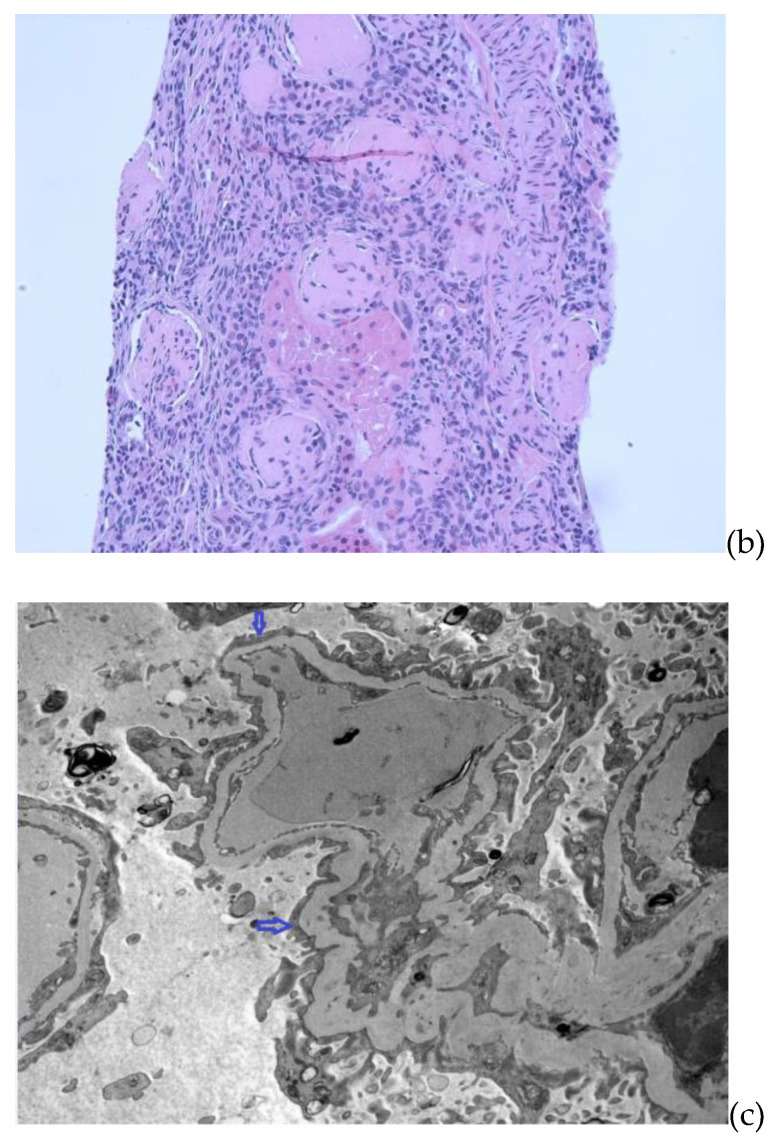
(**a**) Renal biopsy. Light microscopy (H&E) showing a glomerulus with sclerosis in majority of the glomerular capillary tuft, associated with podocyte hyperplasia and foam cells. (**b**) Renal biopsy. Light microscopy (H&E) showing global glomerulosclerosis in the glomeruli with interstitial inflammation in the areas of tubular atrophy and interstitial fibrosis. (**c**) Renal biopsy. Electron microscopy with arrows showing focal areas of podocyte foot process effacement.

Serum creatinine over the next few days increased to 1.8 mg/dL (Schwartz estimated glomerular filtration rate 32 mL/1.73 m^2^/min). Serum intact parathyroid hormone was 217 pg/mL (normal: 12–88 pg/mL). Nephrotic range proteinuria persisted, but the spot urine protein to creatinine ratio decreased to values ranging from 5 to 7 after addition of lisinopril. A percutaneous renal biopsy performed a week after the initial presentation showed evidence of FSGS with severe tubular atrophy and interstitial fibrosis. There was partial foot process effacement (Figure 1a–c). Genetic testing for FSGS showed heterozygous mutations in *ACTN4*, *INF2*, and *KANK1* and homozygous mutation in *NUP93* by next generation sequencing. Owing to inherited gene mutations and the likelihood of steroid resistance, she was not treated with steroid or immunosuppressive agents. Hypertension was managed with lisinopril, amlodipine, and labetalol with stabilization of blood pressures. Antiplatelet agent was added. Iron therapy was started for mild iron deficiency anemia (IDA). Her oxygen saturation returned to normal at the time of discharge and polycythemia resolved without the need for phlebotomy. She subsequently progressed to end stage renal disease (ESRD) ten months after the initial presentation and was started initially on chronic hemodialysis followed by peritoneal dialysis. She received a deceased donor renal transplant four months after being on dialysis with no occurrence of recurrent FSGS during her most recent follow-up two months post-transplant. Her maintenance immunosuppression consisted of tacrolimus, mycophenolate, and prednisone.

## 4. Discussion

EPO is a glycoprotein produced mainly by interstitial cells in the peritubular capillary bed of the kidney [9]. It is produced in response to hypoxia and its synthesis is controlled by hypoxia-inducible transcription factors. The EPO binds to the EPO receptor on the surfaces of immature erythroid cells and stimulates signaling cascades (such as JAK2) for proliferation, differentiation, and anti-apoptosis. In hypoxic conditions, the increased number of circulating red blood cells helps provide adequate oxygen delivery to the tissues [10].

Erythrocytosis is a condition of increased total red blood cell mass and can be primary (polycythemia vera) or secondary. Primary erythrocytosis occurs secondary to primary abnormality of the erythroid precursor cells [11]. Secondary erythrocytosis with increased serum EPO can occur in chronic hypoxic states such as pulmonary diseases, cardiac shunts, sleep apnea, renal artery stenosis, post-renal transplant erythrocytosis, cystic renal disease, ESRD, and hydronephrosis. It can also occur in association with renal cell carcinoma, hepatocellular carcinoma, cerebellar hemangioblastomas, and pheochromocytomas [1]. Serum EPO level is a reliable diagnostic test that should be performed in patients with absolute erythrocytosis [12]. In polycythemia vera, the serum EPO level is suppressed given that the increased red cell production occurs from the abnormal clone in the bone marrow. However, in secondary erythrocytosis, the serum EPO level is increased as a response to hypoxia or anemia, or due to pathologic autonomous EPO production, or from exogenous EPO administration [13].

Nephrotic syndrome is one of the most common glomerular diseases in children [14]. It is manifested by nephrotic range proteinuria, edema, and hypoalbuminemia. About 20% are steroid-resistant (SRNS) and can rapidly progress to ESRD [14]. About 60% of SRNS is due to FSGS, which is a leading cause of early onset CKD in children [15]. Anemia in NS is a common finding. Some of the proposed mechanisms of anemia in NS are altered iron and transferrin homeostasis due to urinary loss, increased urinary loss of EPO, vitamin B12 and copper deficiency, and usage of angiotensin converting enzyme inhibitors for the proteinuria [16].

A multicenter study of 297 patients with NS showed that NS was not associated with anemia in men, but it did tend to decrease hemoglobin levels in women [17]. Vaziri et al. studied 26 adult patients with NS and observed that serum EPO level was inappropriately lower in these patients. This was attributed at least partly to the urinary loss of EPO as the patients with NS had significant amounts of EPO detection in the urine as compared with none in control subjects without NS [18]. This lower serum EPO level potentially could contribute to anemia in NS patients or compound the problem in those with concurrent renal insufficiency with already diminished EPO production. EPO has a molecular weight of 30 Kda [10], hence a significant amount of this might be lost in the urine in actively nephrotic patients. This study also found a significant reduction in the hemoglobin concentration in the NS group compared with the control group [18]. Kemper et al. in their study of 42 children with steroid-sensitive NS showed that serum EPO levels were not different between relapse and remission patients [19]. Similarly, there was no difference in hemoglobin and ferritin levels between relapse and remission groups. However, the serum iron, TIBC, and transferrin levels were significantly reduced and serum soluble transferrin receptor was significantly increased in the relapse group as compared with the remission group. The authors concluded that the upregulation of the soluble transferrin receptor might be important in preventing the development of IDA during the active nephrotic state [19].

In another study, Feinstein et al. studied 19 children with NS who developed anemia before the deterioration of kidney function [20]. They concluded that nephrotic patients have EPO deficiency with a blunted response to anemia and that the EPO deficiency is amenable to exogenous EPO therapy. Patients with IDA without NS have a very high EPO level, while NS patients with IDA have only a very slight rise in EPO level. NS patients with anemia had increased EPO levels as compared with NS patient without anemia, but the increase was significantly less as compared with anemic patients without NS. However, unlike other studies, this study showed that there was no detectable urinary loss of EPO. Hence, urinary loss was not the cause of EPO deficiency as per this study. Moreover, in the experimental model of nephrosis, it has been shown that there is a blunted serum EPO response to hypoxia along with a marked rise in urinary EPO excretion [21]. Our patient had only mild iron deficiency with iron saturation of 18% at the time of presentation, had erythrocytosis, and the serum EPO level was normal. Urine level of EPO was not measured in our patient.

There are several reports of erythrocytosis described in patients with NS secondary to conditions such as membranous nephropathy, FSGS, and minimal change disease, among others [2,22,23]. Some of the proposed mechanisms of erythrocytosis in these patients are increased EPO production due to renal ischemia/hypoxia from thromboembolism or increased sensitivity to EPO, abnormal feedback regulation of erythrocytosis, and EPO-like substance of extra renal origin [22]. Our patient presented with low arterial blood oxygen saturation initially, hence hypoxia could have caused the erythrocytosis. Moreover, nephrotic range proteinuria and hypoalbuminemia can lead to renal interstitial edema and hypoxia [2]. The possible mechanisms of hypoxia-induced EPO secretion are thought to be due to activation of the renin angiotensin II system, leading to increased sodium reabsorption from proximal tubules, increased oxygen consumption, and hence stimulation of oxygen-sensitive receptors and the secretion of EPO [24]. ACE inhibitors are known to be useful in the treatment of erythrocytosis, mainly in the post-renal transplant period; in our patient, its use could have caused resolution of erythrocytosis in addition to decreasing the nephrotic range proteinuria and thereby reducing the renal hypoxia [2]. In our patient, the serum EPO level was normal. This could be because of the fact that the patient also had concomitant nephrotic range proteinuria, which probably led to secondary erythrocytosis from renal hypoxia. This eventually caused a normal and not elevated serum EPO level as the patient might have had increased excretion of EPO in the urine. However, given that the urine EPO level was not available, it remains a speculation and needs to be studied further in future studies. Hence, increased sensitivity to EPO was the most plausible explanation for the erythrocytosis in our patient.

The limitations of our study include unavailability of red cell mass and urine EPO level.

## 5. Conclusions

Secondary erythrocytosis can occur in patients with NS due to renal hypoxia. However, serum EPO level may not be elevated as a result of increased urinary EPO excretion. Hence, alongside the serum level, urinary EPO level may be an useful test in these patients for better understanding of the pathophysiology of this association.

## Figures and Tables

**Table 1:** Laboratory values of hemoglobin and spot urine protein and creatinine.

	Hemoglobin (gm/dL)	Spot Urine Protein to Creatinine Ratio
First admission	17	14.6
D2 of presentation	16.5	Not available
D4 of presentation	15.6	11.03
D7 of presentation	15.4	6.2
D30 of presentation	13.7	Not available
D60 of presentation	12.3	
6 months after presentation	11.5	10.2
At the initiation of dialysis	9.5	Not available
At the time of renal transplant	8.5	Not available
D1 of renal transplant	9.0	0.36

## Data Availability

Not applicable.
